# Penetration of left and right atrial wall and aortic root by an Amplatzer atrial septal occluder in a nine year old boy with Marfan syndrome: Case report

**DOI:** 10.1186/1749-8090-3-25

**Published:** 2008-05-06

**Authors:** Florian Loeffelbein, Christian Schlensak, Sven Dittrich

**Affiliations:** 1 Clinic of Pediatric Cardiology, Children's University Hospital, Freiburg, Mathildenstrasse 1, D-79106 Freiburg, Germany; 2 Department of Cardiovascular Surgery, University Hospital, Freiburg, Hugstetter Strasse 55, D-79106 Freiburg, Germany

## Abstract

**Background:**

To describe complications associated with Amplatzer septal occluders in a patient with Marfan syndrome

**Case presentation:**

A nine-year-old boy with Marfan syndrome and a 22 mm atrial septal defect (ASD) was treated successfully by interventional closure of his ASD by placing a 24 mm Amplatzer septal occluder. Follow up examinations showed a good result but an increasing enlargement of aortic root, so the patient was scheduled for operation. Intraoperative findings showed a perforation of the left atrial roof and the non-coronary sinus by penetration of the occluder device as well as penetration into the right atrial wall. The occluder was resected, the ASD was closed and the aortic sinus was reconstructed using a Dacron patch.

**Conclusion:**

We describe the first case of a patient with Marfan syndrome and an interventional closure of an ASD. Due to alterations of the connective tissue, as it is typical for patients with Marfan syndrome, the Amplatzer occluder probably perforated adjacent structures more easily as in non-affected individuals. Amplatzer occluders should be used with caution and follow up examinations should be performed in short intervals.

## Background

Marfan syndrome is a relatively common genetic disorder with an estimated prevalence of 1: 3,000 to 1:10,000. It is most often caused by mutations in the *FBN1 *(Fibrillin) gene [1,2]. Patients typically present with signs and symptoms related to alterations of their connective tissue including tall stature, recurrent back pain, pectus excavatum, dural ectasia, subluxation of the lens, aneurysm of the aortic root with subsequent aortic regurgitation, and aneurysms of the ascending, descending, or thoracic aorta which can result in rupture and death.

Marfan syndrome is an autosomal dominant disorder with the onset of symptoms occurring in the first decade of life. Nearly 50 percent of patients have to undergo aortic surgery in their lifetime resulting in reconstruction or replacement of the aortic root or total of this vessel's parts [3]. In addition, the occurrence of septal defects in patients with Marfan syndrome is not common.

## Case presentation

**Figure 1:**
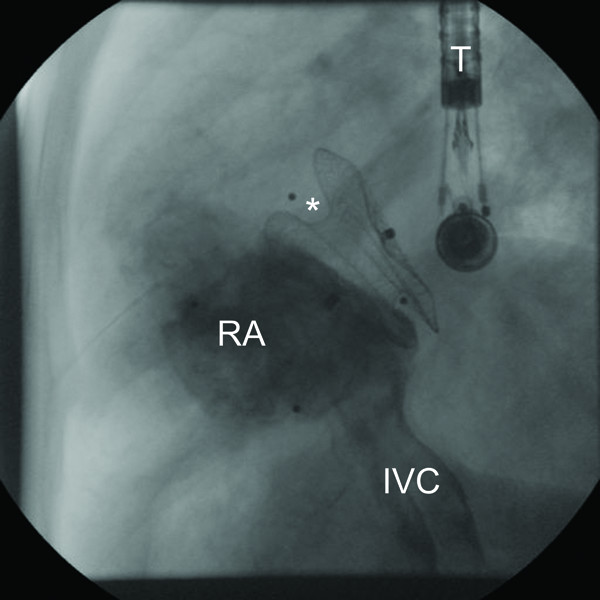
**Lateral fluoroscopy after interventional closure of ASD**: Contrast media is injected in the IVC showing the right atrium. Amplatzer Occluder in position of former ASD bracing from aortic root (asterisk). T: transesophageal probe; IVC: inferior vena cava; RA: right atrium.

We report a nine-year-old boy with an atrial septal defect of secundum type (ASD II) and Marfan syndrome fulfilling the Ghent criteria, i.e. aortic root enlargement, mitral regurgitation and prolaps, pectus excavatum and an elevated arm-span-height-ratio. After informed consent was obtained from the parents, the ASD was treated successfully by interventional closure of the defect with an Amplatzer occluder at the age of 4 (Figure 1). Native defect length in TEE was 17 mm. Transcatheter sizing with a 27 mm Amplatzer sizing-balloon catheter (waisted balloon technique) showed a stretched defect-size of 22 mm. As the transesophageal echo revealed a small aortic rim (3–4 mm), but sufficient margins of the defect to the atrial roof, the caval veins, the coronary sinus and the pulmonary veins, we at this past years chose an oversized Amplatzer device (24 mm) for implantation. After implantation, the occluder typically braced from the aortic root. The follow up ultrasound controls revealed a good result with permanent closure of the septal defect and the device in loco typico. At this time, the aortic root was enlarged to a diameter of 24 mm. Over a period of 2 years, growth of the aortic root to 30 mm was recognized. At this time the diameter exceeded the 95th percentile, and therefore the patient was scheduled for aortic root repair.

**Figure 2:**
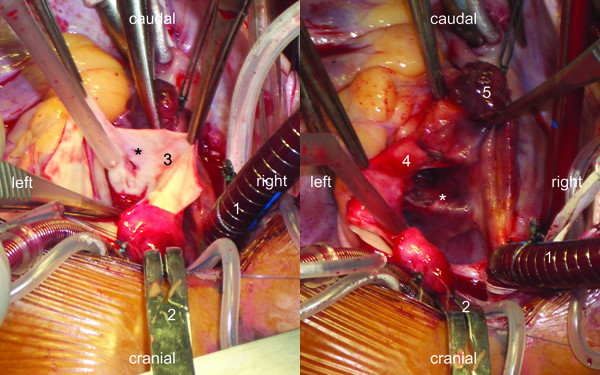
**Overhead view of operating field**: a: Intraaortic view: 1: venous canula; 2: aortic clamp; 3: inside of aortic wall; asterisk: penetration defect of the non-coronary sinus. b: Intraatrial view: 1: venous canula; 2: aortic clamp; 4: ascending aorta; 5: right atrial appendage; asterisk: septal device and penetration defect of the left atrial roof.

From the surgeon's point of view, the Amplatzer septal device had penetrated from the left atrium into the non-coronary sinus of the aortic root with subsequent aneurysmatic ballooning (Figure 2a and 2b). Also, a covered perforation of the left atrial roof was observed and the right atrial disk of the occluder had penetrated into the roof of the right atrium without perforation. The original atrial septal defect was in a central position with an intact superior rim. In light of these findings, the closure device was removed and the septal defect directly closed. Perforated areas were reconstructed by pericardium and the affected sinus was replaced by a Dacron patch.

## Conclusion

To our knowledge this is the first description of aortic root perforation by a septal occluder in a patient with Marfan syndrome. There are only a few cases that report destruction of aortic segments after placement of closure devices in patients with patent foramen ovale (PFO), patent ductus arteriosus (PDA) or ASD [4-7]. Perforation is a rare event, occurring in approximately 0.1% of all cases, indicating closure devices are generally safe. The mechanism of perforation is not well understood but it might be related to the absence or presence of the anterosuperior and posteroinferior rims of the atrial septum where the closure device is fixed in position [8,9]. Interestingly perforations are observed uniquely in the anterosuperior wall and the adjacent aorta [10].

In patients with an altered connective tissue, the reason for dislocation of closure devices might be different. Thus far, we know that the affected protein Fibrillin in Marfan syndrome, plays a crucial role in maintaining the structure of the connective tissue. With the loss of appropriate structure and therefore rigidity, artificial devices may breach and alter the tissue more easily than in non-affected individuals.

Interventionally applied stents that are used to treat thoracic aortic aneurysms in patients with Marfan syndrome [11] are possibly less harmful. Shear forces on the aortic wall are reduced significantly and the vessels are protected from these. Closure devices in septal walls move constantly and may be subjected to greater shearing forces. This can result, as shown in our patient, in deterioration of clinical findings, i.e. augmentation of aortic root's diameter, penetration of adjacent structures, dislocation of the device and the need for early heart or aortic surgery.

Patients with Marfan disease may be at elevated risk for penetration of Amplatzer devices. As long as it is not possible to estimate the individual risk by molecular methods, and the mechanism of penetration is poorly understood, in our opinion, the use of septal occluders should be considered with caution. Even though there are a number of alternative occluders such as the Helex and the Sideris Patch, that may be less rigid, reports of their use in patients with Marfan syndrome are nonexistent. Complication rate, deterioration, and the need of reoperation may occur at an extended level. Thus follow up examinations in those patients should be applied in short intervals. Taking in account these considerations, we would prefer surgery.

## Competing interests

The authors declare that they have no competing interests.

## Authors' contributions

FL collected all data available from the patient and drafted the manuscript including figures and artwork. CS performed the surgical procedures. SD carried out the follow up studies at the Clinic of Paediatric Cardiology. He helped to draft the manuscript, participated in its design and coordination, and revised it critically for important intellectual content.

All authors read and approved the final manuscript.
